# Molecular Understanding of Electrochemical–Mechanical Responses in Carbon-Coated Silicon Nanotubes during Lithiation

**DOI:** 10.3390/nano11030564

**Published:** 2021-02-24

**Authors:** Chen Feng, Shiyuan Liu, Junjie Li, Maoyuan Li, Siyi Cheng, Chen Chen, Tielin Shi, Zirong Tang

**Affiliations:** 1 State Key Laboratory of Digital Manufacturing Equipment and Technology, School of Mechanical Science and Engineering, Huazhong University of Science and Technology, Wuhan 430074, China; cfeng@hust.edu.cn (C.F.); shyliu@hust.edu.cn (S.L.); chenchen_@hust.edu.cn (C.C.); tlshi@hust.edu.cn (T.S.); 2 Shenzhen Institute of Advanced Electronic Materials, Shenzhen Institutes of Advanced Technology, Chinese Academy of Sciences, Shenzhen 518055, China; lijunjie_88@163.com; 3 State Key Laboratory of Materials Processing and Die & Mold Technology, School of Materials Science and Engineering, Huazhong University of Science and Technology, Wuhan 430074, China; limaoyuan@hust.edu.cn; 4 School of Mechanical Engineering and Electronic Information, China University of Geosciences, Wuhan 430074, China; chengsiyi@cug.edu.cn

**Keywords:** carbon nanotube, silicon nanotube, inner hole, lithiation performance, electrochemical-mechanical response, molecular dynamics

## Abstract

Carbon-coated silicon nanotube (SiNT@CNT) anodes show tremendous potential in high-performance lithium ion batteries (LIBs). Unfortunately, to realize the commercial application, it is still required to further optimize the structural design for better durability and safety. Here, the electrochemical and mechanical evolution in lithiated SiNT@CNT nanohybrids are investigated using large-scale atomistic simulations. More importantly, the lithiation responses of SiNW@CNT nanohybrids are also investigated in the same simulation conditions as references. The simulations quantitatively reveal that the inner hole of the SiNT alleviates the compressive stress concentration between a-Li*_x_*Si and C phases, resulting in the SiNT@CNT having a higher Li capacity and faster lithiation rate than SiNW@CNT. The contact mode significantly regulates the stress distribution at the inner hole surface, further affecting the morphological evolution and structural stability. The inner hole of bare SiNT shows good structural stability due to no stress concentration, while that of concentric SiNT@CNT undergoes dramatic shrinkage due to compressive stress concentration, and that of eccentric SiNT@CNT is deformed due to the mismatch of stress distribution. These findings not only enrich the atomic understanding of the electrochemical–mechanical coupled mechanism in lithiated SiNT@CNT nanohybrids but also provide feasible solutions to optimize the charging strategy and tune the nanostructure of SiNT-based electrode materials.

## 1. Introduction

Silicon has long been identified as a highly promising anode material for next-generation lithium-ion batteries because of its high theoretical specific capacity of 3579 mAh/g (≈10 times the capacity of conventional graphite) for forming Li_4.4_Si [1,2,3]. However, the high capacity results in tremendous volume expansion (≈320%) during lithiation, leading to mechanical degradation such as cracks, pulverization, and delamination followed by rapid capacity fade, low current/Coloumbic efficiency, and short cycle life [4,5,6]. Accordingly, various silicon nanostructures such as nanoparticles, nanowires, and nanotubes have been applied to battery anodes to facilitate stress relaxation and avoid mechanical fractures [7,8,9,10,11,12,13,14,15].

Although remarkable performance has been achieved by various nanostructured silicon anodes, there are continuous demands in tuning those nanostructures for better resistance to morphological instability and performance degradation during the lithiation process. For achieving the goals, it is extremely required to figure out the electrochemical–mechanical evolution mechanism of those nanostructures during lithiation/delithiation cycles, which can provide fundamental understanding to explore feasible strategies for designing improved silicon anodes [16,17,18,19,20,21]. For the crystalline silicon nanowires and nanopillars, the two-phase lithiation and anisotropic expansion (preferential expansion along 〈110〉 directions) during lithiation have been widely acknowledged [22,23,24]. Furthermore, the self-limiting lithiation behavior caused by lithiation-induced stress has also been demonstrated [25,26]. These studies indicate that the lithiation kinetics and morphological evolution of silicon nanostructures have complex chemo-mechanical mechanisms, which are closely related to the crystal orientation, geometry shape, structural size, and so on. Based on the knowledge of the fundamental lithiation mechanism, some attempts have been made to optimize the design for more rational silicon anodes, where silicon nanotubes (SiNT) are believed to better accommodate a large volume change during lithiation due to the additional empty internal space [27,28]. Moreover, silicon nanotubes are also capable of promoting lithium ion diffusion because of the extra inner surfaces accessible to the electrolyte [29,30]. Encapsulating the silicon nanostructures with self-supporting and void-containing carbon materials (core–hollow shell nanohybrids) has been demonstrated as an effective strategy to tackle the structural and interfacial instability issues [31,32,33,34,35,36]. However, those carbon-coated nanostructures also induce new disadvantages, such as structural fracture in silicon nanotubes, the constraint effect of coating layers, and the barrier function of void spaces in core–hollow shell nanohybrids [37,38,39]. Therefore, it is necessary to further explore optimal nanostructures of carbon-coated silicon nanotubes for better durability and higher safety. However, some fundamental understanding of the lithiation kinetics and morphological evolution of SiNT-based anodes still remains unclear. 

So far, most experimental techniques are not sufficiently sensitive to simultaneously track the chemical, structural, and mechanical evolution of the nanostructures. In this context, the computational models based on the classical finite strain theory and nonlinear diffusion approximation are used to provide an interpretation of stress generation in lithiated silicon anodes recently. For instance, Wang et al. performed a finite element simulation to investigate the hoop stress evolution of lithiated 〈111〉 silicon nanotubes with different original sizes [20], while Shi et al implemented a chemomechanical model to simulate the lithiation processes in both the C-coated SiNW and Si-C yolk-shell nanoparticles (NPs) [40]. Even though the continuum modeling is helpful to understand the stress evolution and degradation mechanism, this method still needs a priori assignment of the chemical strain, which is difficult to measure directly. Those models ignore the valuable structural information on the atomic scale. Considering the quantitative analysis, actual atomic-level physics, and longer timescales, the molecular dynamics (MD) simulation based on a reactive force field (ReaxFF) is developed to investigate the lithiation/delithiation responses of silicon-based nanostructures, which can provide a multiscale approach to simulate chemical reactions such as bond formation and breaking in nanoscale systems [41,42]. So far, the electrochemical–mechanical evolution has been widely investigated in silicon nanowires, nanoparticles, and their coated nanohybrids by ReaxFF-MD, such as Si@Al_2_O_3_ [43,44], Si@SiO_2_ [45], Si@CNT [39], and Si@graphene [46]. However, there is still no systematical atomic simulation about SiNT and its coated nanohybrids. In addition, in view of the fact that simulation studies usually adopt different model sizes and reaction temperature, there is a lack of comparative analysis of lithiation responses in SiNT-based samples and other Si-based samples under the same simulation conditions. The lack of systematical and comparative study strongly restricts the development of the SiNT-based electrode materials.

In the present work, we provided a systematical and sufficient investigation of the lithiation behaviors in bare SiNT and its carbon-coated nanohybrids (SiNT@CNT) using ReaxFF-MD simulations. The lithiation responses of SiNW and its carbon-coated nanohybrids (SiNW@CNT) are also investigated as references. By quantitatively analyzing the differences between these nanohybrids in lithiation performance (lithiation rate and capacity) and structural information (concentration distribution and stress evolution), the influences of inner hole structure and contact mode between the core and shell are demonstrated. Our insightful perspectives enrich the fundamental research on electrochemical–mechanical evolution mechanism of SiNT and help to design better SiNT-based anodes for LIBs.

## 2. Simulation Methods

We performed the molecular dynamics (MD) simulations using large-scale atomic/molecular massively parallel simulator (LAMMPS) with a reactive force field (ReaxFF) [47], which have been demonstrated as a powerful and reliable method to simulate the lithiation process of various Si-C hybrid nanostructures. Here, the ReaxFF parameters for Li-Si-O-C systems are used [46]. All simulations in this work were carried out in the canonical ensemble (NVT) at the temperature of 500 K using the velocity Verlet algorithm with a time step of 0.2 fs. The temperature of 500 K is well below the melting temperature of Li-Si alloys; this temperature can accelerate the lithiation and exclude the influence of the excessive temperature [48].

**Figure 1:**
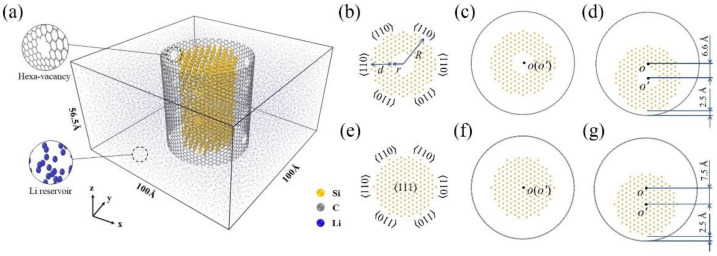
(**a**) Illustration of the reactive force field (ReaxFF)-molecular dynamics (MD) simulation of the lithiation process. Schematic illustration of the cross-sectional shape of (**b**) silicon nanotubes (SiNT) sample, (**c**) concentric carbon-coated silicon nanotube (SiNT@CNT) sample, (**d**) eccentric SiNT@CNT sample, (**e**) SiNW sample, (**f**) concentric SiNW@CNT sample and (**g**) eccentric SiNW@CNT sample.

For the bare silicon nanotube sample, the initial crystalline structure is 〈111〉 oriented with a wall thickness (*d*) of 10.6 Å, inner radius (*r*) of 5.3 Å, and outer radius (R) of 15.9 Å (cross-sectional shape is shown in Figure 1b) containing 2006 Si atoms; its cross-section has six equivalent 〈110〉 directions. The interplanar spacing along the 〈110〉 direction is larger than those along other directions, providing a dominant ion channel for lithium implantation, which realized the isotropic volume expansion of Si core along the radial direction during simulation. The *r*/*R* = 1/3 is believed to be an appropriate ratio for the best structural stability in silicon nanotubes during lithiation [20]. For the coated silicon nanotube samples, armchair single-walled carbon nanotube (CNT) coatings with a radius of 25 Å are added, and hexa-vacancies (about 5% vacancy concentrations) are introduced to facilitate the Li atoms penetration through hexagon rings. Li atoms penetrating through carbon vacancies in the CNT layer tend to preferentially diffuse at the interface between the CNT layer and Si surface rather than penetrate into the Si core directly [46]. Therefore, these defects would not cause inhomogeneous lithiation in the Si nanotube. In addition, the different contact modes between silicon nanotubes and the CNT shell are considered. As shown in Figure 1d, off-center distance, the distance between the axis of the silicon nanotube (*O’*) and the axis of the CNT shell (*O*), is introduced to construct two different coated samples, a concentric structure (off-center distance is 0 Å, Figure 1c) and an eccentric structure (off-center distance is 6.6 Å along the negative y direction, Figure 1d). For the lithiation process, these three samples are surrounded by about 24000 Li atoms in 3D periodic simulation boxes at the dimension of 100 Å ×100 Å × 56.5 Å; the length of the SiNT samples is the same as that of the z dimension of the simulation box, and the lithiation process is spontaneous due to the large negative heat of mixing. All these configurations simulate an infinitely long silicon nanotube and its CNT-coated nanohybrids lithiated along the radial directions.

To deeply figure out the lithiation responses of silicon nanotubes, the lithiation behaviors of silicon nanowire (SiNW) and its CNT-coated nanohybrids (SiNW@CNT) are also investigated as references by similarly immersing in Li reservoir. The initial crystalline silicon nanowire is 〈111〉 oriented with a radius of 15 Å (cross-sectional shape is shown in Figure 1e) containing about 2000 Si atoms. The configurations of CNT coatings and the Li reservoir are the same as those of silicon nanotube samples. Even though the void-containing SiNW@CNT nanostructures have been investigated in our previous study [39], combining the lithiation responses of SiNT and SiNW, and comparing the differences between them can provide remarkably valuable information for revealing the chemo-mechanical lithiation mechanism.

## 3. Results and Discussion

### 3.1. Morphological Evolution and Lithiation Performance of Silicon Nanotubes

**Figure 2:**
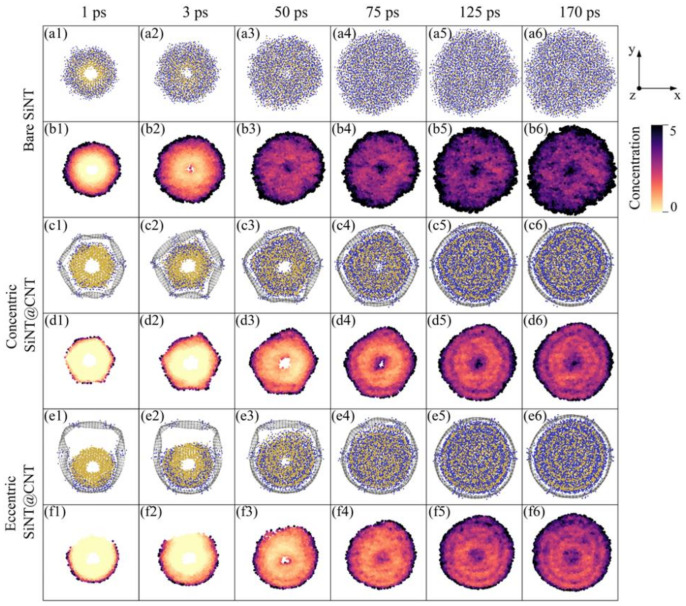
Morphological evolution. The cross-section snapshots for atomic structural evolution at a specific simulation time during the lithiation of (**a1**–**a6**) Bare SiNT, (**c1**–**c6**) Concentric SiNT@CNT, and (**e1**–**e6**) Eccentric SiNT@CNT. The lithium concentration maps at a corresponding simulation time of (**b1**–**b6**) Bare SiNT, (**d1**–**d6**) Concentric SiNT@CNT, and (**f1**–**f6**) Eccentric SiNT@CNT.

**Figure 3:**
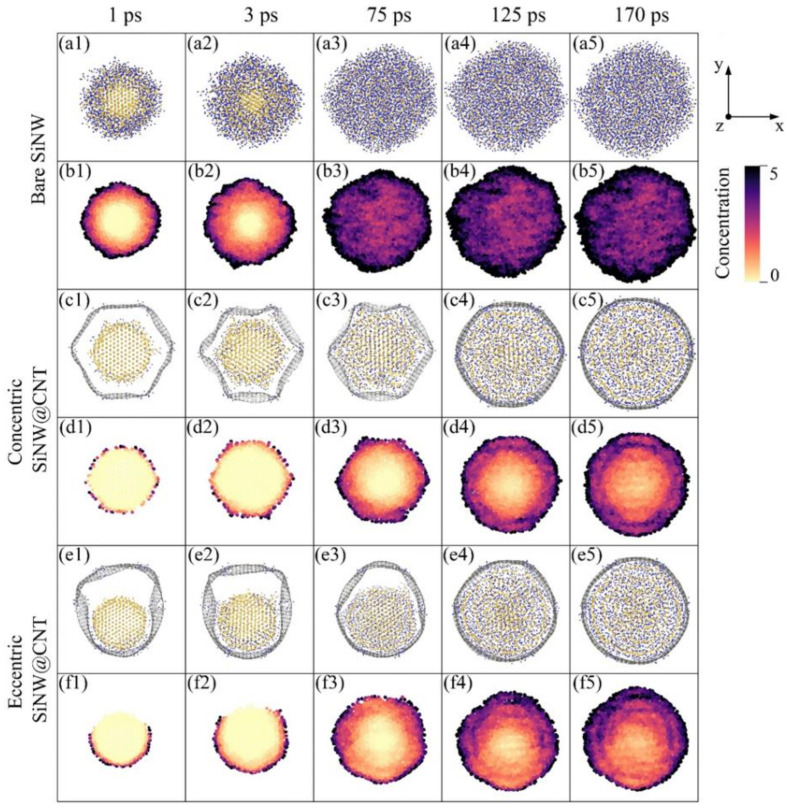
Morphological evolution. The cross-section snapshots for atomic structural evolution at specific simulation time during lithiation of (**a1**–**a5**) Bare SiNW, (**c1**–**c5**) Concentric SiNW@CNT, and (**e1**–**e5**) Eccentric SiNW@CNT. The lithium concentration maps at a corresponding simulation time of (**b1**–**b5**) Bare SiNW, (**d1**–**d5**) Concentric SiNW@CNT, and (**f1**–**f5**) Eccentric SiNW@CNT.

The morphological evolution of SiNT, concentric SiNT@CNT, and eccentric SiNT@CNT samples can be investigated by tracking the atomic structure evolution during lithiation, as shown in Figure 2. The morphological evolution of SiNW samples is shown in Figure 3. For clarity, the Li atoms in reservoir are represented as transparent. For the lithiation process of the bare SiNT sample, Li atoms flow into the SiNT along the radial direction at a very fast lithiation rate at the initial stage, leading to a dramatic volume expansion. The dominant volume expansion seems similar to an isotropic expansion within the cross-section, which could be explained by the preferential lateral expansion in the 〈110〉 direction. The interplanar spacing along the 〈110〉 direction is larger than those along the other directions, providing a dominant ion channel for lithium implantation, thus leading to more significant expansion in the 〈110〉 direction. Meanwhile, the inner hole of SiNT appeared to degrade suddenly with the Li atoms penetrating into the inner hole. The CNT-coated SiNT shows different lithiation responses. For the concentric SiNT@CNT sample, at the initial stage of lithiation, Li atoms firstly penetrate through the hexa-vacancies and cover the inner surface of the CNT shell, which retards the Li atoms penetrating into the SiNT. Then, the Li flows radially into the silicon, while the following lithiation process is similar to that of the bare SiNT sample. Obviously, the existence of the CNT coating decreases the lithiation rate, further retarding the morphological evolution, while the inner hole structure appears to remain for a longer period in SiNT@CNT. For the eccentric SiNT@CNT sample, due to the eccentric structure shortening the distance between the outer surface of the SiNT and the inner surface of the CNT, the Li atoms could penetrate into the silicon more quickly than the concentric sample. As a result, the structural evolution of the inner hole is affected by this eccentric structure, resulting in that the inner hole structure of the eccentric sample disappears earlier than that of concentric sample. In addition, the eccentric structure causes an asymmetrical lithiation pattern.

Although the atomic structural snapshots to some extent reflect the morphological evolution of these samples, the continuous lithium implantation would make it difficult for the lithiated silicon structure hard to be observed directly, especially the inner hole structure. To further quantify the morphological information, we measured the volume change, Li capacity evolution, and local Li concentration map (shown in Figure 2) in bare SiNT, concentric SiNT@CNT, and eccentric SiNT@CNT samples during lithiation, respectively. The details of the Li concentration map of SiNW samples are given in Figure 3. To get the relevant information, the outer surface of SiNT was first defined by averaging the coordination along the radial direction of the outermost 10% of Si atoms. The volume of the lithiated sample was measured according to the outer surface and its ratio with the volume of the initial sample as the volume changes. Li concentration was defined by the numbers of Li and Si atoms inside the outer surface, as *x* in Li*_x_*Si, and the Li capacity (C) was calculated by the following formula:

(1) $$C=\frac{x\times e\times N_{A}}{1\times 28\;g/\mathrm{mol}}$$

where $e=1.602\times 10^{-19}C,\;N_{A}=6.02\times 10^{23}{\;\mathrm{mol}}^{-1}$. The local Li concentration map was obtained by measuring the normalized ratio between the number of Li and Si atoms inside a 3 Å cutoff of each atom. 

**Figure 4:**
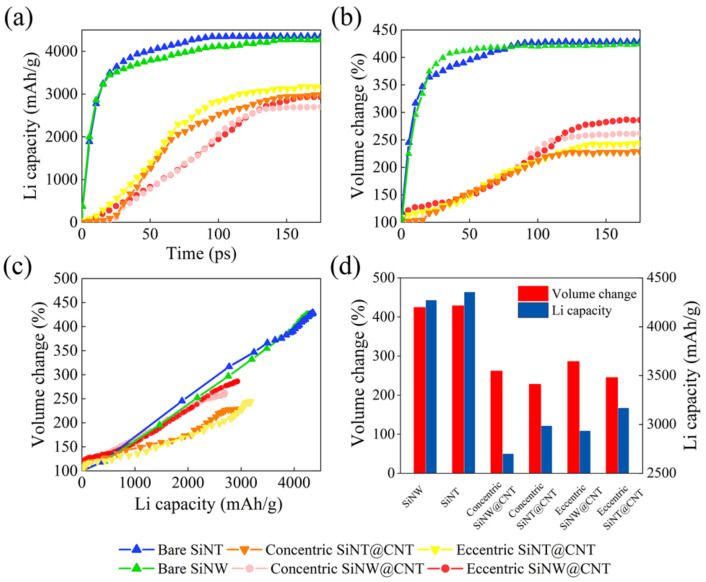
Lithiation performances of SiNT, silicon nanowires (SiNW), and their carbon nanotube (CNT)-coated nanohybrids. (**a**) Li capacity with lithiation time, (**b**) Volume change with lithiation time, (**c**) Volume change versus Li capacity during lithiation, and (**d**) Volume change and Li capacity of the final lithiated products.

To deeply understand the lithiation performance of the SiNT and its CNT-coated nanohybrids, the information of volume and Li capacity of the SiNW and its CNT-coated nanohybrids are also added in Figure 4a–c as references, and the volume and Li capacity of these final lithiated products are summarized in Figure 4d. For the bare SiNT and SiNW samples, at the initial stage of the lithiation (about 20–75 ps), the volume of SiNT is smaller than that of SiNW, but the Li capacity is higher than that of SiNW. This can be explained by the local Li concentration map in these two samples (as shown in Figure 2(b1–b4) and Figure 3(b1–b3)). The SiNW sample has an obvious two-phase lithiation behavior, a core–shell lithiated pattern consisting of a pristine c-Si core (light yellow) and a lithiated amorphous Li*_x_*Si shell (dark red), but the existence of an inner hole in SiNT mediates the two-phase lithiation. Specifically, before the phase boundary reached the inner hole surface, SiNT also showed a two-phase lithiation behavior (as shown in Figure 2(b1,b2)). With the lithiation proceeding, the phase boundary gradually migrated to the unlithiated core region and reached the inner hole of SiNT. Finally, the inner hole makes the core region become another Li-rich region (Li concentration is up to 3.75, dark red is shown in Figure 2(b3,b4)). This seems like a three-phase lithiation behavior, full-lithiated core/no full-lithiated region/full-lithiated shell. As a result, at the medium stage of lithiation, the inner hole structure enhances the Li capacity and relieves volume expansion in some cases. It should be pointed out that the Li concentration of the no full-lithiated phase is also up to 3.0–4.0 (Figure 2(b5,b6)) at the end of the lithiation, which caused the Li capacity and volume of full lithiated SiNW to be nearly the same as those of full lithiated SiNW (Figure 4a,b).

The two-phase lithiation also exists in the concentric SiNW@CNT and eccentric SiNW@CNT samples, as shown in Figure 3, but due to the constraint effect of the CNT shell, the Si core still remains no full-lithiated at the end of lithiation. Similarly, the concentric SiNT@CNT and eccentric SiNT@CNT samples have three-phase lithiation behavior, and the no full-lithiated region still exists at the end of lithiation. Therefore, the SiNT@CNT samples show different Li capacity evolution and volume change with SiNT@CNT samples. Both the lithiation rate (Figure 4a) at the medium stage and the Li capacity of the final lithiated product (Figure 4d) of SiNT@CNT are higher than that of SiNW@CNT. The final lithiated concentric SiNT@CNT has 2984.3 mAh/g and the final lithiated eccentric SiNT@CNT has 3166.8 mAh/g, but the lithiated concentric and eccentric SiNW@CNT samples only have 2697.2 and 2933 mAh/g, respectively. However, the final volume expansion of SiNT nanohybrids are lower than that of SiNW nanohybrids. Obviously, the existence of an inner hole structure enhances the lithium capacity and alleviates the volume expansion.

Regardless of SiNT or SiNW samples, the eccentric structure shows its considerable superiority. On the one hand, the eccentric structure can accelerate the lithiation rate by shortening the void space between the silicon core and carbon shell, which is directly revealed by the Li capacity evolution at the initial lithiation stage (Figure 4a), while the Li capacity of eccentric samples are higher than those of concentric samples in the first 30 ps. On the other hand, the eccentric structure can enhance the Li capacity of the final products (Figure 4d). Most importantly, this enhancement in SiNW@CNT (≈9%, from 2697.2 to 2933 mAh/g) is higher than that in SiNT@CNT (≈6%, from 2984.3 to 3166.8 mAh/g), which can be explained by the stress-regulated effects discussed in Section 3.3. 

Consequently, for the bare structure, an inner hole structure can enhance the Li capacity and relieve the volume expansion in the medium stage of lithiation. For the CNT-coated structure, the inner hole not only enhances the lithiation rate at the medium stage but also enhances the Li capacity in the final lithiated product. In addition, using an eccentric structure can further promote the lithiation rate and Li capacity in void-involved Si@CNT nanohybrids.

### 3.2. Structural Evolution of Inner Hole 

According to the above quantitative analyzation, the inner hole plays a key role in the morphological evolution and lithiation performance. However, the continuous lithium implantation would cause the inner hole structure hard to be observed directly in the atomic structural snapshots and Li concentration maps. Thus, we measured the boundary of the inner hole to obtain more quantitative structural information. The boundary of the inner hole was first defined by averaging the coordination along the radial direction of the innermost 10% of Si atoms; then, the volume of the inner hole was measured according to this inner surface.

**Figure 5:**
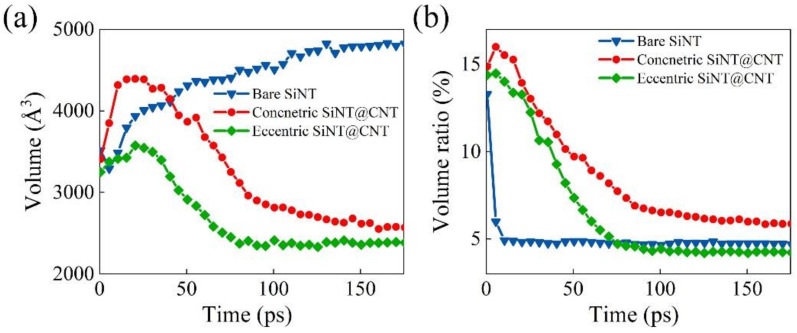
Evolution of inner holes in bare SiNT, concentric SiNT@CNT, and eccentric SiNW@CNT. (**a**) The volume of inner hole changes with time and (**b**) volume ratio (the volume of inner hole/total volume) as a function of time in the lithiation process.

As revealed in Figure 5, for the bare SiNT, the volume of the inner hole increases steadily during lithiation time (from 3516.9 to 4822 Å^3^, ≈37.1%), due to the dramatic volume expansion of SiNT (up to 428%) during lithiation, while the volume ratio of the inner hole gradually reduces (from 13.3% to 4.7%), which shows that the inner hole of SiNT undergoes much less expansion relative to the outsides. This indicates that the inner hole structure in SiNT has mechanical stability to some extent, which is in accordance with the previous experiment observation and further verifies that the 〈111〉 oriented SiNT with a thickness (d) and outer radius (R) ratio of 2/3 has good fracture resistance. Good structural stability can keep the enhancement of the inner hole on lithiation performance lasting for several lithiation/delithiation cycles. As a contrast, for the concentric SiNT@CNT sample, the volume of the inner hole increases from 3406.7 to 4284.2 Å^3^ (≈25.8%) at the initial stage of lithiation, but it decreases with the lithiation further proceeding and finally reduces to 2569.1 Å^3^ (≈24.6%). Meanwhile, due to the constraint effect of the CNT shell, the volume expansion of SiNT is restricted, which results in the volume ratio of the inner hole increasing from 14.8% to 15.3% at the initial stage. With the lithiation proceeding, the volume ratio finally decreases to 6.0%. A similar volume and volume ratio change of the inner hole can be observed in an eccentric SiNT@CNT sample, but the maximum volume (3574.7 Å^3^) and final volume (2358.5 Å^3^) of the inner hole in the eccentric sample are lower than those in the concentric sample during lithiation, and the volume ratio (4.2%) of the inner hole in the eccentric sample is lower than that in the concentric sample at the end. Clearly, it can be seen that both the coating layer and the contact model affect the morphological evolution of the inner hole. On the one hand, the lithiated a-Li*_x_*Si phase is restricted by the coating layer, and in turn, it expands toward the inner hole surface, which destroys the stability of the inner hole structure. On the other hand, regulating the contact mode to form an eccentric structure promotes the lithium penetration to the inner region, further accelerating the degradation of the inner hole structure. Consequently, in full lithiation condition, the structural stability of bare SiNT is better than that of SiNT@CNT. It is necessary to further optimize the SiNT@CNT anodes to enhance their durability.

For the bare SiNT structures, previous in situ observation and finite element simulation [20] have revealed that most fractures occur on the outer surface of SiNT, and tensile stress concentration determines the anisotropic fractures at the outer surfaces. The bare SiNT with d/R=2/3 has the lowest maximum hoop stress and consequently the lowest fracture ratio. The fracture ratio increases as the wall thickness or inner radius (r) increases. These results confirmed that tuning the size of the hole and the relationship between wall thickness and its diameter is very important for better resistance to failure. However, for the SiNT@CNT hybrids, the protective function of CNT coatings mediates the lithiation-induced stress distribution, which effectively relieves the outer-surface fractures of SiNT but in turn causes inner-surface contraction (as shown in Figure 5a). Therefore, enhancing the structural stability of the inner hole is extremely important in the SiNT@CNT structure. Here, according to our quantitative analysis of the inner hole, optimizing the charging strategy would be a useful method to alleviate the great contraction of the inner hole to enhance the stability. For example, if the lithiation is kept for 75 ps, in the concentric SiNT@CNT sample, the volume of the inner hole only decreases from 3406.7 to 3249.9 Å^3^, but the Li capacity has been up to 2127.8 mhA/g, which is about 5.7 times larger than that of the carbonaceous anodes (372 mAh/g for LiC_6_). It implies that mediating the charging degree can effectively circumvent the incompatibilities between lithiation performance and structural stability in SiNT@CNT-based anodes. In addition, optimizing the inner-hole surface structure would also help relieve the contraction of the inner hole and consequently enhance the structural stability. For example, construct another C coating layer on the inner surface to form a C@Si@C sandwich structure; this structure shows no mechanical/microstructural damage during lithiation/delithiation cycling [11].

### 3.3. Stress-Regulated Effects during Lithiation

It is well recognized that the stresses generated in the silicon-based materials regulate the kinetics of chemical reactions and potential response [25,49]. The mechanism by which the tensile/compressive stress facilitates/retards the diffusion of Li atoms may cause the inhomogeneous growth of the lithiated phase, leading to the nonuniform distribution of morphology and Li ion concentration [17,45,50,51,52,53]. To further study this, the stress evolution is quantitively investigated. The residual stress was obtained by averaging the atomic radial stresses of all atoms in the concentric shell between *r* and r+δr, where *r* is the radial distance from the center of SiNT. The radial stress ($\sigma_{r}$) is calculated according to Equation (2).

(2) $$\sigma_{r}=\sigma_{xx}\cos^{2}\theta \;+\;2\sigma_{xy}\sin\theta \cos\theta \;+\;\sigma_{yy}\sin^{2}\theta$$

**Figure 6:**
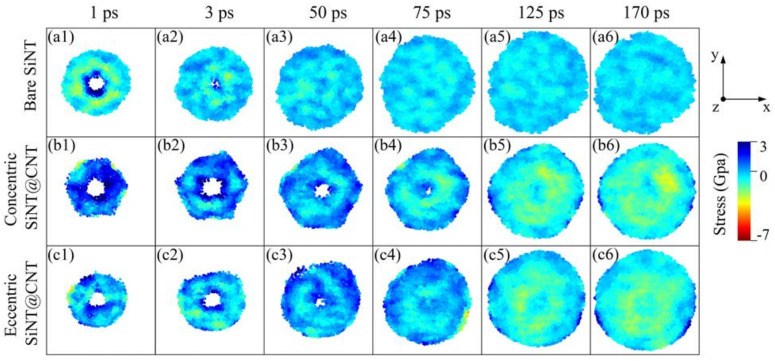
Stress-distribution maps of (**a1**–**a6**) bare SiNT, (**b1**–**b6**) concentric SiNT@CNT, and (**c1**–**c6**) eccentric SiNT@CNT during lithiation, positive (+) and negative (−) values correspond to tensile and compressive stresses, respectively.

**Figure 7:**
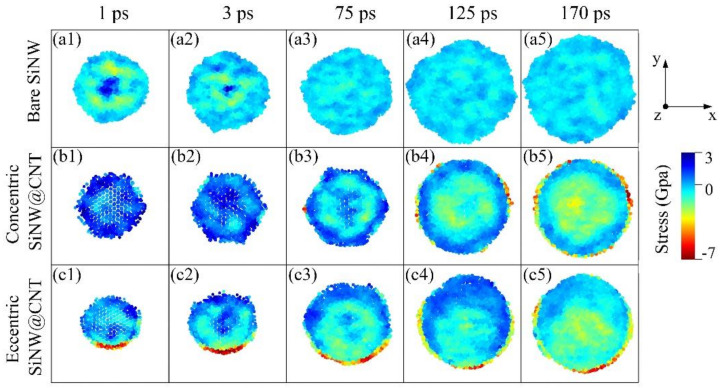
Stress-distribution maps of (**a1**–**a5**) bare SiNW, (**b1**–**b5**) concentric SiNW@CNT, and (**c1**–**c5**) eccentric SiNW@CNT during lithiation, positive (+) and negative (−) values correspond to tensile and compressive stresses, respectively.

Here, the $\sigma_{xx}$, $\sigma_{yy}$, and $\sigma_{xy}$ are the normal stress, normal stress, and shear stress in a Cartesian coordinate system (*x, y, z*), respectively, whose *z*-axis is aligned with the central axis of the nanotube. The θ is an angle between the radial vector and the *x*-axis in the *x-y* plane. The stress distribution maps in the lithiated SiNT are shown in Figure 6. The stress distribution maps of SiNW and its CNT-coated nanohybrids are shown in Figure 7 as references. Comparing these stress distribution maps of SiNT and SiNW samples is extremely helpful to reveal the effects of the inner hole structure and contact mode between core and shell on the morphological evolution and lithiation performance.

Comparing the bare SiNT (Figure 6(a1–a6)) with the bare SiNW (Figure 7(a1–a5)), initially, the tensile hoop stress concentration occurs at the unlithiated core region in SiNW, and the tensile hoop stress concentration occurs at the unlithiated inner hole surface in SiNT; both tensile stress concentrations give rise to the compressive stresses at the lithiated phase boundary. With the lithiation proceeding, the Li atoms penetrate into the core region and the inner hole surface, causing the tensile stress concentrations to disappear and inducing these regions to be subjected to slightly compressive stresses. It can be seen that the stresses distribution and degree of stress concentration are nearly same in bare SiNT and bare SiNW samples, which explains why the lithiation performance of bare SiNT is almost same as that of bare SiNW (Figure 4). More importantly, the morphological evolution of the inner hole (Figure 5) is in good agreement with the stress evolution in SiNT; the tensile stress concentration at the inner hole surface causes the expansion of the hole at the initial stage. Then, the slight compressive stress makes the change of inner hole become steady. As a result, the inner hole structure in SiNT has good mechanical stability.

Comparing the SiNT@CNT samples (Figure 6(b1–b6,c1–c6)) with SiNW@CNT samples (Figure 7(b1–b5,c1–c5). For the concentric SiNW@CNT sample, due to the constraint effect of the CNT shell, the compressive stress concentration occurs at the outer surface of SiNW, while the maximum compressive stress is up to 6.6 Gpa during the whole lithiation process. Moreover, the stress distribution shows a symmetrical pattern, causing the symmetrical lithiation, which confirms the stress-regulated effects. For an eccentric SiNW@CNT sample, the eccentric structure caused a higher degree of compressive stress concentration (up to 7.6 Gpa) at the connected region between lithiated a-Li*_x_*Si and the CNT shell, and the highly asymmetrical stress distribution results in highly asymmetrical lithiation. However, the stress evolutions in SiNW@CNT samples are quite different from those in SiNW@CNT samples. On the one hand, the existence of an inner hole alleviates the compressive stress concentration caused by the CNT shell. The degree of compressive stress at the outer surface is obviously lower than that in SiNW@CNT; the maximum compressive stress is only 3.1 Gpa in concentric SiNT@CNT and 4.2 Gpa in eccentric SiNT@CNT during lithiation, which caused a faster lithiation rate and higher Li capacity than those of the SiNW@CNT samples (as shown in Figure 4). Moreover, the degree of compressive stress concentration near the inner surface is much higher than that in bare SiNT. As a result, the inner hole structure would shrink dramatically with lithiation proceeding, which verifies the investigation mentioned in Section 3.2 in which the inner hole expands first and then shrinks. On the other hand, the existence of an inner hole weakens the influence of the contact mode. The eccentric structure still gives rise to the asymmetrical stress distribution at the initial stage of lithiation, further causing an asymmetrical lithiation pattern in SiNT@CNT, but the stress distribution and lithiation pattern in the final lithiated product show no obvious symmetrically. This response is due to that shown in Figure 6(c3); the lithiation reaction front arrives at the inner hole more quickly inside the bottom region, causing the compressive stress to take place in the lower side of the inner hole. Meanwhile, the upper side of the inner hole is still under tensile stress, which gives rise to an asymmetrical stress distribution on the inner surface. Moreover, the inner hole structure provides empty spaces for lithiated a-Li*_x_*Si phase movement to accommodate the mismatch of stress distribution, resulting in a rise in the structural instability of inner hole. It also demonstrates the investigation in Section 3.2 that the volume of the inner hole in eccentric SiNT@CNT shrinks faster than that of the concentric one (as shown in Figure 5).

In general, it can be seen that compared with the SiNW@CNT nanohybrids, the inner hole structure exactly promotes the lithiation performance of the SiNT@CNT nanohybrids. In contrast to the high capacity, the primary challenge of coated-SiNT electrode materials should be how to alleviate the mechanical degradation of the inner hole, which can ensure the enhancement of the inner hole in as many lithiation/delithiation cycles as possible. Based on our insightful investigations, using a partial charging strategy can avoid the inner hole shrinking dramatically. In addition, the roots of structural degradation are the compressive stress concentration and the mismatch of the stress distribution at the inner hole surface; thus, further optimizing the design of the inner hole structure is required. It should be pointed out that the electrochemical–mechanical evolution mechanism revealed in our work is not only suitable for the SiNT-based nanohybrids, but it is also suitable for other inner hole-containing electrode materials.

## 4. Conclusions

In conclusion, we performed a series of large-scale MD simulations based on a ReaxFF force field to investigate the electrochemical–mechanical responses of SiNT@CN nanohybrids during lithiation, and the influences of the inner hole structure and contact mode between the core and shell are deeply explored by comparing with the lithiation behaviors of SiNW@CNT nanohybrids. The simulation results reveal that compared with the nanowire structure, the nanotube structure further promotes the Li capacity and lithiation rate, especially in the CNT-coated nanostructures. These enhancements are well explained by the stress-regulated effects; the inner hole alleviates the compressive stress concentration between the lithiated a-Li*_x_*Si and C phases. The effects of the inner hole and contact mode on the structural stability of SiNT are demonstrated. At the initial stage of lithiation, the inner holes of any SiNT nanohybrids undergo tensile hoop stress concentration, leading to volume expansion of holes. With the lithiation proceeding, the inner hole of bare SiNT shows good structural stability due to the no stress concentration at the inner surface, and the inner hole undergoes much less expansion than the outsides. Meanwhile, the inner hole of concentric SiNT@CNT undergoes dramatic shrinkage due to the compressive stress concentration, and the inner hole of eccentric SiNT@CNT is deformed due to the mismatch of stress distribution. Based on this quantitative electrochemical and mechanical information at the nanoscale, executing partial charging to control the dramatic shrink is proved as an efficient strategy to enhance the structural stability of inner hole structure. These findings can open avenues for optimizing the structural design of various SiNT-based electrode materials for high-capacity and long-cycle life LIBs.

## Data Availability

The data presented in this study are available on request from the corresponding authors.
